# The AGC Kinase SsAgc1 Regulates *Sporisorium scitamineum* Mating/Filamentation and Pathogenicity

**DOI:** 10.1128/mSphere.00259-19

**Published:** 2019-05-29

**Authors:** Yixu Wang, Yi Zhen Deng, Guobing Cui, Chengwei Huang, Bin Zhang, Changqing Chang, Zide Jiang, Lian-Hui Zhang

**Affiliations:** a State Key Laboratory for Conservation and Utilization of Subtropical Agro-Bioresources, South China Agricultural University, Guangzhou, China; b Guangdong Province Key Laboratory of Microbial Signals and Disease Control, Integrative Microbiology Research Centre, South China Agricultural University, Guangzhou, China; Carnegie Mellon University

**Keywords:** AGC kinase, *Sporisorium scitamineum*, cAMP, filamentous growth

## Abstract

*Sporisorium scitamineum* is the fungal pathogen causing severe sugarcane smut disease that leads to massive economic losses globally. *S. scitamineum* invades host cane by dikaryotic hyphae, formed after sexual mating of two haploid sporidia of opposite mating type. Therefore, mating/filamentation is critical for *S. scitamineum* pathogenicity, while its molecular mechanisms remain largely unknown. The AGC (cyclic AMP [cAMP]-dependent protein kinase 1 [protein kinase A {PKA}], cGMP-dependent protein kinase [PKG], and protein kinase C [PKC]) kinase family is a group of serine/threonine (Ser/Thr) protein kinases conserved among eukaryotic genomes, serving a variety of physiological functions, including cell growth, metabolism, differentiation, and cell death. In this study, we identified an AGC kinase, named SsAgc1 (for *S. scitamineum* Agc1), and characterized its function by reverse genetics. Our results showed that SsAgc1 is critical for *S. scitamineum* mating/filamentation and pathogenicity, and oxidative stress tolerance under some circumstances. Transcriptional profiling revealed that the SsAgc1 signaling pathway may control expression of the genes governing fungal mating/filamentation and tryptophan metabolism, especially for tryptophol production. We showed that tryptophan and tryptophol could at least partially restore *ssagc1*Δ mating/filamentation. Overall, our work revealed a signaling pathway mediated by AGC protein kinases to regulate fungal mating/filamentation, possibly through sensing and responding to tryptophol as signal molecules.

**IMPORTANCE** The AGC signaling pathway represents a conserved distinct signaling pathway in regulation of fungal differentiation and virulence, while it has not been identified or characterized in the sugarcane smut fungus *Sporisorium scitamineum*. In this study, we identified a PAS domain-containing AGC kinase, SsAgc1, in *S. scitamineum*. Functional analysis revealed that SsAgc1 plays a regulatory role on the fungal dimorphic switch.

## Abstract (precis)

The AGC signaling pathway represents a conserved distinct signaling pathway in regulation of fungal differentiation and virulence, while it has not been identified or characterized in the sugarcane smut fungus *Sporisorium scitamineum*. In this study, we identified a PAS domain-containing AGC kinase, SsAgc1, in *S. scitamineum*. Functional analysis revealed that SsAgc1 plays a regulatory role on the fungal dimorphic switch.

## INTRODUCTION

During the pathogenic life cycle of the sugarcane smut fungus *Sporisorium scitamineum*, three distinct cell morphologies and lifestyles are documented, namely, haploid sporidium (yeast-like), dikaryotic hypha, and diploid teliospore. The yeast-like sporidia consisting of two opposite mating types, e.g., *MAT-1* and *MAT-2*, are nonpathogenic. The fusion of two compatible sporidia, or sexual mating, results in formation of a pathogenic dikaryotic hypha, which is capable of infecting the host plant canes (1). The dikaryotic hypha grows within the sugarcane, along with bud meristem, and a diploid teliospore is formed by fusion of two compatible nuclei, followed by a round of meiosis that occurs during teliospore germination that gives rise to four haploid sporidia (2). The molecular mechanism of sexual mating and filamentation is mostly unknown in *S. scitamineum*, although it has been extensively investigated in the model organism Ustilago maydis, the causal fungus of corn smut (3–6). The conserved mating-type loci, *a* locus and *b* locus, are present in *S. scitamineum.* Functional study of the *a* locus genes in *S. scitamineum* has been limited to *MFA2* (7), and the role of *b* locus in fungal mating has been validated through deletion analysis (1).

Quorum sensing (QS) in bacteria plays a role in coordinating cell density-dependent gene expression, including virulence genes in bacterial pathogens (8, 9). Studying fungal QS has recently started. In Candida albicans or Saccharomyces cerevisiae, two aromatic alcohols tyrosol and tryptophol have been reported as filamentation-promoting QS molecules (10–12), and both are degradation products of aromatic amino acids in the Ehrlich pathway (13). Feedback regulation of S. cerevisiae morphology by tryptophol is mediated by the cyclic AMP (cAMP)/protein kinase A (PKA) signaling pathway, integrating sensing of nitrogen availability (11). However, there have not been any reports of any signaling pathway regulating the production of aromatic alcohols essential for C. albicans filamentation. Another fungal QS molecule, farnesol, identified in C. albicans as a negative regulator of hyphal formation, was shown to be regulated by a two-component system (14). The Cek1 mitogen-associated protein kinase (MAPK) involved in filamentous growth in C. albicans has been shown to be regulated by the QS molecule farnesol through suppression of Sho1 (a transmembrane adaptor protein)-dependent Cek1 phosphorylation (15). Our preliminary bioinformatic analysis identified several *S. scitamineum* orthologs of C. albicans Cek1 MAPK signaling components, but the functional study is lacking.

MAPK and cAMP/PKA signaling pathways, which are highly conserved in eukaryotic organisms, perceive and respond to a variety of external or internal signals for the regulation of cell growth, development, stress response, and pathogenicity (16–19). They have been reported to control a range of physiological processes, including fungal development and infection, in various pathogenic fungi such as Magnaporthe oryzae (20), Fusarium graminearum (21), U. maydis (22), C. albicans (23), Aspergillus flavus (24), and Cryptococcus neoformans (16). The AGC kinase family includes cAMP-dependent protein kinase 1 (PKA), cGMP-dependent protein kinase (PKG), and protein kinase C (PKC), which share sequence similarities in their catalytic domains and are widely conserved among eukaryotic genomes (25). Although AGC kinases are important regulators of cell growth, metabolism, and cell death, much remains to be learned about their molecular functions and targets in plant or fungal organisms (26).

We recently identified a serine/threonine kinase, annotated as a homolog of Schizosaccharomyces pombe Cek1, from our transcriptome analysis between the wild-type *S. scitamineum* and its *b* locus deletion mutant under mating conditions (27). This serine/threonine kinase belongs to the AGC kinase family and is thus named SsAgc1 (for *S. scitamineum* Agc1). We found that the *SsAGC1* gene is differentially expressed between mating and nonmating *S. scitamineum* cultures (27), indicative of a potential function in fungal mating/filamentation. In this study, we generated and characterized the *ssagc1*Δ mutant in mating/filamentation and disease symptom development. We found that SsAgc1 was required for *S. scitamineum* mating/filamentation, and transcriptional profiling demonstrated that SsAgc1 regulates expression of genes controlling fungal mating/filamentation and encoding enzymes for tryptophan metabolism. Exogenous addition of tryptophan or tryptophol could restore the defective mating/filamentation in the *ssagc1*Δ mutant. SsAgc1 is also required for *S. scitamineum* pathogenicity, possibly due to its function in dikaryotic hyphal formation and/or oxidative stress tolerance. We infer that SsAgc1 may participate in a signaling pathway to promote fungal mating/filamentation.

## RESULTS

### Identification of an AGC kinase in *S. scitamineum*.

**FIG 1:**
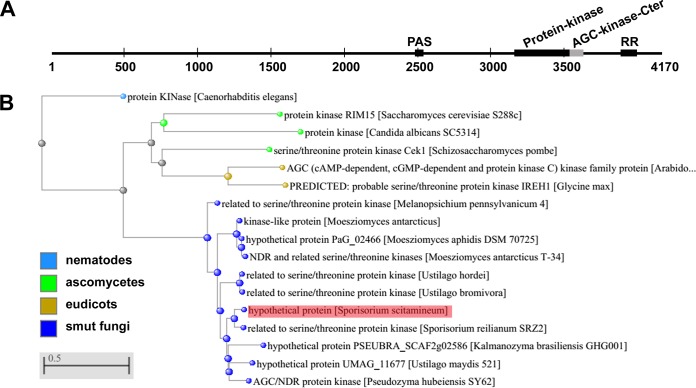
Domain and phylogenetic analyses of the *S. scitamineum* AGC kinase SsAgc1. (A) Schematic diagram of the domain structure of the SsAgc1 protein. Individual domains of SsAgc1 are represented by black or gray boxes, with the domain names given above the boxes. The PAS domain is the Per-Arnt-Sim domain, usually found in a protein acting as a signal sensor. The response regulatory (RR) domain contains a predicted phosphorylation site at 3943D residue. The domains of SsAgc1 were identified with interPro-Scan (http://www.ebi.ac.uk/interpro/). (B) Phylogenetic analysis of the deduced amino acid sequences of SsAgc1. The phylogenetic tree is constructed using the neighbor-joining phylogeny of COBALT, the constraint-based multiple alignment tool (https://www.ncbi.nlm.nih.gov/tools/cobalt/re_cobalt.cgi) (50). The position of SsAgc1 in the phylogenetic tree is indicated by the pink highlighting. The NCBI:protein accession numbers of all the aligned kinases are given in parentheses (species names are given in brackets as in the figure): SsAgc1 (CDR87150.1), related to serine/threonine protein kinase [Sporisorium reilianum SRZ2] (CBQ71694.1), hypothetical protein UMAG_11677 [Ustilago maydis 521] (XP_011388072.1), AGC/NDR protein kinase [Pseudozyma hubeiensis SY62] (XP_012190076.1), related to serine/threonine protein kinase [Ustilago hordei] (CCF53636.1), related to serine/threonine protein kinase [Ustilago bromivora] (SAM79781.1), kinase-like protein [Moesziomyces antarcticus] (XP_014657970.1), related to serine/threonine protein kinase [Melanopsichium pennsylvanicum 4] (CDI52366.1), hypothetical protein PaG_02466 [Moesziomyces aphidis DSM 70725] (ETS62728.1), NDR and related serine/threonine kinases [Moesziomyces antarcticus T-34] (GAC76453.1), hypothetical protein PSEUBRA_SCAF2g02586 [Kalmanozyma brasiliensis GHG001] (XP_016292461.1), serine/threonine protein kinase Cek1 [Schizosaccharomyces pombe] (SpCek1, NP_588310.1), protein kinase RIM15 [Saccharomyces cerevisiae S288c] (ScRim15, NP_116620.1), AGC (cAMP-dependent, cGMP-dependent and protein kinase C) kinase family protein [Arabidopsis thaliana] (NP_201037.1), PREDICTED: probable serine/threonine protein kinase IREH1 [Glycine max] (XP_006587460.1), protein KINase [Caenorhabditis elegans] (NP_001255552.1), and protein kinase [Candida albicans SC5314] (CaRim15, XP_720337.1).

A serine/threonine kinase-encoding gene was previously identified as a significantly (*P* ≤ 0.05) differentially expressed gene in a nonmating and nonfilamentous mutant compared to the wild-type strain (27); therefore, it may serve a function in fungal mating/filamentation. The reads obtained by RNA sequencing with the wild-type sample (27) covers the gene as annotated by NCBI (https://www.ncbi.nlm.nih.gov/nuccore/1003330579; locus_tag “SPSC_00276”) and skipped the region annotated as intron. Therefore, we followed the NCBI annotation of this gene for prediction of its encoding product. The predicted polypeptide (NCBI:protein accession no. CDR87150.1) consists of 4,170 amino acid residues, with a molecular weight (MW) of 435.72 kDa and pI of 6.15. As it belongs to the AGC (cAMP-dependent, cGMP-dependent, and protein kinase C) kinase family, we named it SsAgc1. SsAgc1 protein contains a PAS domain (amino acids [aa] 2486 to 2546), a protein kinase domain (aa 3162 to 3561) harboring an ATP-binding domain (aa 3168 to 3304), the kinase active site (aa 3307 to 3502) with two activation loop (A-loop) motifs (aa 3303 to 3313 and 3457 to 3465), an AGC-kinase-Cter domain (aa 3562 to 3655), and a response regulatory (RR) domain of the CheY-like superfamily (aa 3893 to 4010) (Fig. 1A).

Serine/threonine protein kinase homologs were identified using a BLASTP search against the nonredundant protein sequence (nr) database with SsAgc1 protein sequence as bait. The top 10 hits (sequence coverage of >80% and identity of >60%) were all from smut fungi. We also performed BLink search using SsAgc1 protein sequence and selected the top hits from ascomycetes, including S. pombe, S. cerevisiae, and C. albicans, from plants, including Arabidopsis thaliana and Glycine max, and from the nematode Caenorhabditis elegans, to generate the phylogenetic tree together with the top 10 most similar AGC kinases from smut fungi. The reconstructed phylogenetic tree is shown in Fig. 1B. SsAgc1 was clustered with the serine/threonine protein kinase from Sporisorium reilianum SRZ2, and most closely related to the kinases of three other smut fungi, Ustilago maydis, Ustilago hordei, and Ustilago bromivora. The serine/threonine protein kinases from two plant species were clustered in the other clade and related to ascomycetes. The nematode kinase clustered separately in an outgroup (Fig. 1B). No signature motif of the AGC groups (28) was identified in the SsAgc1 sequence, and the related serine/threonine kinases in the phylogenetic tree were classified as members of the other group AGC kinase family, including S. pombe Cek1 (SpCek1), S. cerevisiae Rim15 (ScRim15), and C. albicans Rim15 (CaRim15) (28), suggesting that the SsAgc1 protein is a member of the “other type” AGC kinases.

The amino acid sequence conservation between SsAgc1 and SpCek1 or ScRim15 is low and restricted in the protein kinase domain. Thus, the function of SsAgc1 may not be conserved with these two ascomycetous orthologs. The function of this AGC family protein has not been characterized in smut fungi. We generated the *ssagc1*Δ mutants in *S. scitamineum* and characterized them in sexual mating, dikaryotic hyphal formation, and pathogenicity.

### SsAgc1 is essential for *S. scitamineum* mating/dikaryotic hyphal formation.

**FIG 2:**
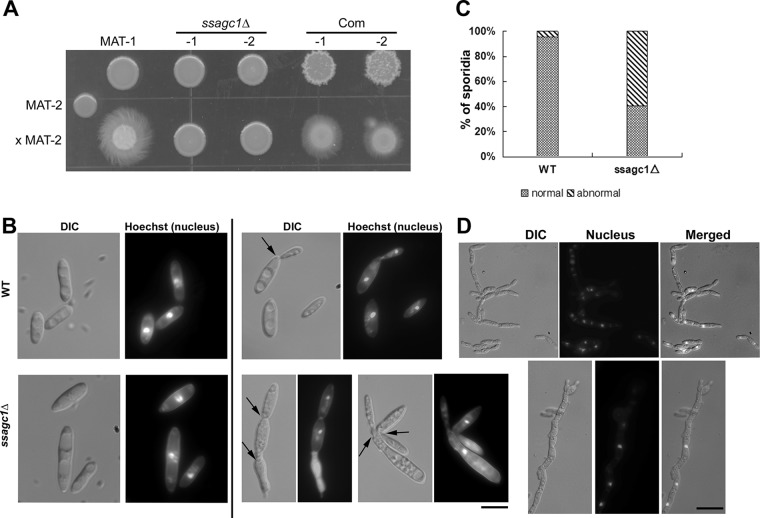
SsAgc1 is essential for *S. scitamineum* mating/filamentation. (A) Mating assay with the sporidia of wild-type (WT) *MAT-1*, two *SsAGC1* deletion mutants (*ssagc1*Δ -1 and -2), and two *SsAGC1* complementation (Com) strains (-1 and -2), with the wild-type *MAT-2* sporidia. (B) Hoechst (nucleus) staining with *MAT-1* (wild type) and the *ssagc1*Δ sporidia. Images were taken with an Axio Observer Z1 microscope equipped with an sCMOS camera, using a DAPI filter. Images on the left panel were showing sporidia of normal morphology and contain one nucleus per cell. Images on the left panel represent normal sporidia, while images on the right were representative of abnormal sporidia/pseudohypha, with one nucleus per septum (denoted by black arrows) but the dividing cells failed to separate. DIC, differential interference contrast. Bar = 20 μM. (C) Quantification of percentage of sporidia of normal or abnormal (psuedohyphal) morphology in the WT or *ssagc1*Δ mutant, based on the microscopic documentation as shown in panel B. *n* ≥ 300 for each instance. (D) The *ssagc1*Δ pseudohypha after sexual mating, costained by calcofluor white and Hoechst. Bar = 20 μM.

The *ssagc1*Δ mutants were generated in the wild-type (WT) *S. scitamineum MAT-1* background (see Fig. S1A in the supplemental material), and two independent strains were confirmed by Southern blotting (Fig. S1B). We assessed *S. scitamineum* mating/filamentation by mixing and spotting sporidia of opposite mating type on solid PDA medium. The *in vitro* culture of mixed WT *MAT-1* and *MAT-2* sporidia gave rise to dikaryotic hyphae, and thus, the colonies had a fluffy appearance (Fig. 2A). Mixed cultured WT *MAT-2* with the *ssagc1*Δ mutants, however, displayed obviously reduced filamentous and radial growth (Fig. 2A), although the *ssagc1*Δ sporidial colony was comparable to the WT sporidial colony. Genetic complementation of the *SsAGC1* gene (Fig. S1C) was performed using a truncated fragment encoding the region containing only annotated domains (aa 2082 to 4170) of the SsAgc1 protein, as detailed in Materials and Methods. These genetically complemented strains could exhibit fully restored mating/filamentation, although the sporidial colony of the complemented strains looked different (drier) from that of the WT or *ssagc1*Δ mutants (Fig. 2A). This result confirmed that *SsAGC1* is required for *S. scitamineum* mating/filamentation.

10.1128/mSphere.00259-19.1 FIG S1 Generation and verification of *ssagc1*Δ mutants and genetic complementation strains. (A) Schematic representation of generation of two partially overlapped fragments with HPT-resistant marker (truncated arrowed box) flanked with 5′ and 3′ regions of the *SsAGC1* gene (arrowed box). Homologous recombination between these two fragments and *S. scitamineum* genome results in replacement of the *HPT* gene with the *SsAGC1* gene. The PCR-amplified *HPT* gene fragment and a fragment containing the 3′ region (denoted by scale bar = 1 kb) were used as the probe for DNA gel blot analysis shown in panel B. The positions of restriction enzyme ClaI is depicted in the scheme. (B) Southern blot analysis for confirmation of the *ssagc1*Δ mutants. Genomic DNA from the WT (*MAT-1*) or the indicated transformants was digested with the restriction enzyme ClaI and probed with the 2.0-kb *HPT* fragment or *SsAGC1*-specific probe, respectively. The appearance of the 3.0-kb band detected by *HPT* probe or by the *SsAGC1*-specific probe with the concomitant loss of the wild-type 5-kb *SsAGC1* locus served as a validation of the replacement of *SsAGC1* coding sequence with the *HPT* fragment. (C) Deletion and genetic complementation of SsAGC1 gene was confirmed by quantitative real-time PCR (qRT-PCR). Relative gene expression level was calculated by the −ΔΔ*C_T_* method (51) with *ACTIN* as an internal control. Primers used for transcriptional profiling were listed in Table 1. Asterisks represent significant difference (*P* < 0.05) compared to the WT MAT-1. (D) Mating/filamentation of wild-type *MAT-1* and *ssagc1*Δ mutants, with wild-type *MAT-2*, was assessed on PDA or MM-N solid medium, with or without the addition of 10 mM cAMP. Photographs were taken 3 days postinoculation. Download FIG S1, PDF file, 0.8 MB. Copyright © 2019 Wang et al. 2019 Wang et al. This content is distributed under the terms of the Creative Commons Attribution 4.0 International license.

Interestingly, *ssagc1*Δ mating/filamentation could be restored when cultured under nitrogen-starved conditions (MM-N in Fig. S1D), indicating that nutrient-restricted conditions may promote filamentation even without SsAgc1 function. We further tested the effect of cAMP on *ssagc1*Δ filamentation. The addition of 10 mM cAMP could effectively promote filamentous growth in the mixed culture of *MAT-2* and *ssagc1*Δ (Fig. S1D). Next, we determined the intracellular concentration of cAMP in the wild type and *ssagc1*Δ mutant, with the deletion mutant of *SsUAC1* (29), an ortholog to the *U. maydis* adenylyl cyclase-encoding gene *UAC1* (30), as a positive control. The results showed that out of 10^7^ sporidia, the cAMP concentration of the wild-type strain was 27.87 pmol/ml, while that in the *ssuac1*Δ mutant was 0.74 pmol/ml, confirming that cAMP production was indeed blocked due to the loss of adenylyl cyclase function. However, the cAMP concentration of the *ssagc1*Δ mutant was 25.39 pmol/ml, comparable to that in the wild type. These results indicate that SsAgc1 may function downstream of cAMP or in a parallel pathway in regulating *S. scitamineum* mating/filamentation.

We examined the cell morphology of WT and *ssagc1*Δ mutant by microscopy, after staining nuclei with fluorescent dye Hoechst. Most of the WT sporidia appeared like typical rod-shaped cells, containing one nucleus per cell, as expected (Fig. 2B). In contrast, the *ssagc1*Δ sporidia were frequently seen as elongated, “chain-like” cells that resembled pseudohyphae with one nucleus per septum (Fig. 2B). Such altered cell morphology was likely due to failed cytokinesis. The percentage of such normal/abnormal (pseudohyphal) cell morphology was quantified in both WT and *ssagc1*Δ sporidia (*n* ≥ 300; Fig. 2C), and the results indicated that SsAgc1 kinase was involved in regulation of sporidial cell morphology and/or proper mitosis. We further compared the morphology of dikaryotic hyphae after sexual mating between the WT and the *ssagc1*Δ mutant. The WT dikaryotic hyphae were long and smooth and contained two nuclei per section, as shown in Fig. S2A, which was rarely seen in the *ssagc1*Δ mutant. In most cases, *ssagc1*Δ sporidia mixed with the WT sporidia remained as individual yeast-like cells (Fig. S2B), confirming that sexual mating was blocked due to the loss of SsAgc1 function. We occasionally saw cells in a chain in the WT × *ssagc1*Δ culture (Fig. 2D), but the hyphal morphology was distinct from that of WT dikaryotic hypha formed after sexual mating (Fig. S2A). Costaining with Calcofluor white (for cell wall and septum) and Hoechst (nucleus) confirmed that such structures were pseudohyphae, as they contained only one nucleus per septum (Fig. 2D). Overall, we confirmed that the *ssagc1*Δ mutant was defective in sexual mating and dikaryotic hypha formation, which could be restored by the addition of cAMP.

10.1128/mSphere.00259-19.2 FIG S2 Costained hyphal septa and nuclei in the WT and *ssagc1*Δ mutant. (A) The wild-type dikaryotic hypha after sexual mating, costained by calcofluor white (septa denoted by short bars) and Hoechst (nuclei denoted by arrows). (B) The *ssagc1*Δ sporidia (mixed with wild-type sporidia of the opposite mating type) or pseudohypha after sexual mating, costained by calcofluor white and Hoechst. Scale bar = 20 μM. Download FIG S2, TIF file, 1.2 MB. Copyright © 2019 Wang et al. 2019 Wang et al. This content is distributed under the terms of the Creative Commons Attribution 4.0 International license.

### SsAgc1 regulates tolerance to oxidative stress.

**FIG 3:**
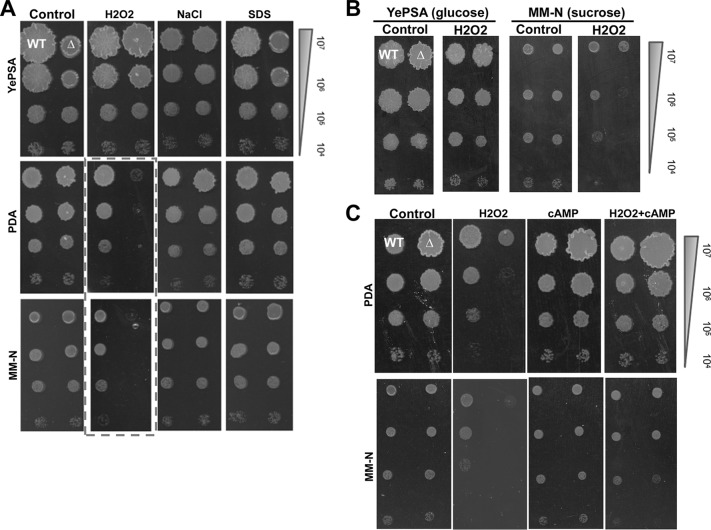
SsAgc1 is required for tolerance to oxidative stress. (A) Serially diluted cells of *MAT-1* (WT) or *ssagc1*Δ mutant (Δ) were spotted onto YePSA, PDA, or MM medium supplemented with H_2_O_2_ (1 mM), NaCl (500 mM), or SDS (0.1 mM). The control is the untreated culture. Images were taken 72 h postinoculation. (B) Carbohydrate swapping between YePSA and MM-N medium to test the effect of carbon source on oxidative tolerance. Serially diluted cells of *MAT-1* (WT) or *ssagc1*Δ mutant (Δ) were spotted onto the modified YePSA (glucose as the sole carbon source) or modified MM-N medium (sucrose as the sole carbon source) supplemented with 1 mM H_2_O_2_. Images were taken 72 h postinoculation. (C) Sporidia from *MAT-1* (WT) and *ssagc1*Δ mutant (Δ) that had been serially diluted were spotted onto solid PDA or MM-N medium alone or supplemented with 1 mM H_2_O_2_ and/or 10 mM cAMP. Images were taken 72 h postinoculation.

Next we tested tolerance of *S. scitamineum* sporidia toward various stressful conditions, including oxidative stress (H_2_O_2_), osmotic stress (NaCl), and cell wall stress (SDS). We noticed that a hypersensitivity response to H_2_O_2_ resulted from *SsAGC1* deletion, specifically in cultures grown on PDA or MM-N but not in the cultures grown on YePSA medium (Fig. 3A). No obvious difference was observed in terms of osmotic stress or cell wall stress resistance (Fig. 3A). We tested the effects of different carbon sources by comparing YePSA (sucrose) and MM-N (glucose) media and did not find any change in the hypersensitivity toward oxidative stress in the *ssagc1*Δ mutant (Fig. 3B), indicating that the tolerance to oxidative stress was regulated by SsAgc1 in a glucose-independent manner. We further assessed the effect of cAMP on oxidative stress tolerance. The results showed that the addition of 10 mM cAMP could effectively restore resistance to 1 mM H_2_O_2_ in the *ssagc1*Δ sporidia, on either PDA or MM-N medium (Fig. 3C), indicating an overlap between the SsAgc1 and cAMP signaling pathways in regulating tolerance to oxidative stress.

Overall, we found that SsAgc1 was required for tolerance to oxidative stress, but it was not directly related to glucose availability as reported for other PAS domain-containing AGC kinases (31, 32).

### SsAgc1 is essential for *S. scitamineum* pathogenicity.

**FIG 4:**
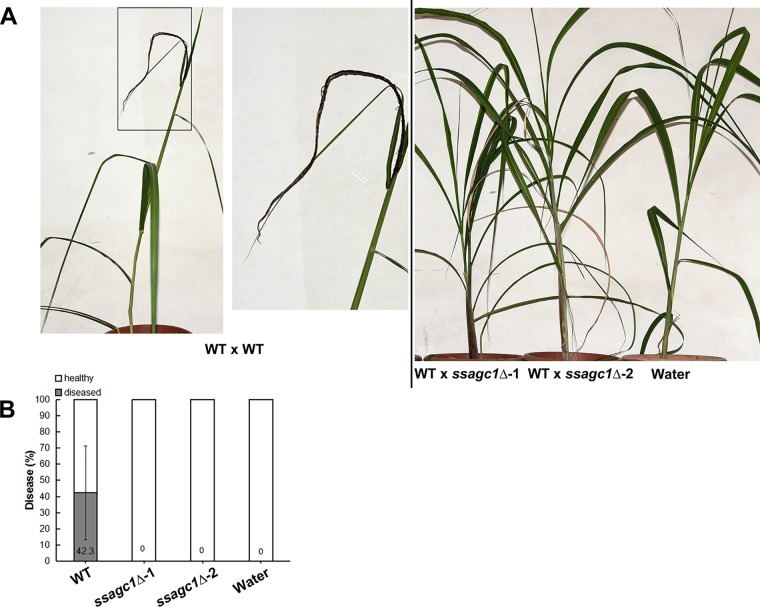
SsAgc1 is required for *S. scitamineum* pathogenicity. (A) Sugarcane variety ROC22 susceptible to *S. scitamineum* was inoculated with mixed fungal sporidia by injecting seedlings at the 5- to 6-leaf seedling stage. (Left) WT control treatment resulted in black “whip” formation 90 to 180 days after injection. The boxed region in the left panel was enlarged to better show the symptoms. (Right) Two independent *ssagc1*Δ mutants (*ssagc1*Δ-1 and *ssagc1*Δ-2) sporidia were mixed with the wild-type *MAT-2* strain (WT) and injected into the sugarcane seedlings. Plants injected with water served as a negative control. Infection assay was performed in three independent repeats, with 5 to 10 plants/inoculation. (B) Bar chart depicting quantification of infection as shown in panel A. Means ± standard errors (error bar) were derived from three independent repeats (*n* ≥ 25).

To determine whether *SsAGC1* is involved in pathogenicity, we inoculated the susceptible sugarcane seedlings with the mixed *S. scitamineum* sporidial cells of opposite mating types in the combinations of WT × WT or WT × *ssagc1*Δ. The sugarcane seedlings inoculated with WT × WT sporidial mixture displayed the characteristic symptoms of “smut whip” emerging from the shoots at 90 to 120 days postinoculation (Fig. 4A). In contrast, seedlings infected by WT × *ssagc1*Δ combination remained healthy through the observed period of 180 days and never developed whip symptom (Fig. 4A and B). Therefore, we conclude that *SsAGC1* is required for *S. scitamineum* pathogenicity, which is likely due to its function in regulating mating/filamentation and/or oxidative stress tolerance.

### SsAgc1 controls expression of the genes governing fungal mating/filamentation and tryptophan metabolism.

To obtain a better understanding of SsAgc1 regulation of *S. scitamineum* mating/filamentation, we performed a transcriptome analysis to identify potential target genes of SsAgc1 signaling, under mating conditions, comparing the WT × WT combination and the WT × *ssagc1*Δ combination. We identified 1,019 differentially expressed genes (DEGs; |log2| ≥ 1 and *P* ≤ 0.05), of which 344 overlapped in the two biological replicates (Fig. S3A and Data Set S1). The pathways enriched with these overlapping DEGs suggest that SsAgc1 may promote transcription of genes involved in tryptophan metabolism (Data Set S1, Fig. S3B, and Fig. S4), as well as MAPK signaling pathway involved in fungal mating (Fig. S5). The *a* locus gene *PRA2*, fungal mating regulator encoding gene *PRF1*, and the genes encoding enzymes involved in tryptophan metabolism were among the DEGs (Data Set S1). We then performed quantitative real-time PCR (qRT-PCR) to verify the differential expression of selected genes in the *ssagc1*Δ mutant compared to the WT, mixed with compatible WT sporidia of the opposite mating type to induce mating/filamentation. These selected genes included *a* and *b* locus genes and transcriptional factor *PRF1* involved in fungal mating/filamentation, and *ARO8*, *ARO9*, *DC*, *TYNA* and *amiE* genes for tryptophol or indoleacetate (IAA) biosynthesis from tryptophan. Our results showed that the *a* locus genes *MFA1*, *PRA1*, and *PRA2* were indeed significantly (*P* ≤ 0.05) downregulated, while the *b* locus gene *bE* or *bW* was not changed significantly (Table 1). *TYNA1* was upregulated (*P* ≤ 0.01), while *amiE* downregulated (*P* ≤ 0.01) in the *ssagc1*Δ mutant during *S. scitamineum* mating/filamentation (Table 1). DEGs were consistently identified by transcriptome analysis and qRT-PCR as listed in Table 1 and schematically illustrated as Fig. S6, suggesting that SsAgc1 may regulate transcription of genes controlling fungal mating/filamentation and tryptophan metabolism in *S. scitamineum*.

**TABLE 1:** Transcriptional profiling of the genes governing fungal mating/filamentation and tryptophan metabolism in WT versus *ssagc1*Δ mutant^*a*^

Gene	Transcriptome						qRT-PCR^*b*^	
	Repl1			Repl2				
	Fold change	*P* value	FDR	Fold change	*P* value	FDR	Fold change	*P* value
***MFA1***							**0.63 ± 0.048**	**0.020**
*MFA2*							0.86 ± 0.036	0.565
***PRA1***				0.002702703	1	1	**0.35 ± 0.020**	**0.000**
***PRA2***	**0.349759202**	**3.68E−55**	**5.57E−54**	**0.242836379**	**3.07E−60**	**7.74E−59**	**0.57 ± 0.006**	**0.046**
*bE*	1.335573203	3.74E−09	1.13E−08	1.044941176	0.477871701	0.591602872	1.09 ± 0.018	0.229
*bW*	0.912618796	0.319386543	0.392941896	0.938679245	0.572072143	0.680661697	0.08 ± 0.010	0.234
***PRF1***	**0.452162971**	**1.0355E−216**	**8.5359E−215**	0.556129612	1.24317E−95	5.58873E−94	**0.53 ± 0.015**	**0.013**
*ARO8*	1.923309385	9.26E−239	8.76E−237	1.71034805	2.90E−129	2.04E−127	1.30 ± 0.117	0.084
*ARO9*	0.526359833	1.52E−17	7.12E−17	0.631093544	2.47E−07	8.95E−07	0.90 ± 0.014	0.121
	0.617305127	1.31E−11	4.57E−11	0.871517447	0.106619846	0.166193215	0.69 ± 0.044	0.885
*TYNA-1*	4.592037229	2.71E−91	7.37E−90	3.484407484	3.10E−13	1.77E−12	1.38 ± 0.012	0.003
*TYNA-2*	0.935560859	0.318924845	0.392441976	0.907796102	0.161804625	0.23816831	0.70 ± 0.019	0.473
***amiE***	**0.381931841**	**5.93E−78**	**1.34E−76**	**0.421607687**	**1.45E−50**	**3.04E−49**	**0.65 ± 0.200**	**0.004**

a The fold change, *P* value, and false-discovery rate (FDR) for two replicates (replicate 1 [Repl1] and 2) are shown for the transcriptome data. Downregulated genes and data are shown in boldface type, while the upregulated gene and data are shown underlined.

b Relative gene expression fold change was calculated by the −ΔΔ*C_T_* method (51) with *ACTIN* as an internal control.

10.1128/mSphere.00259-19.3FIG S3Identification of potential target genes of the SsAgc1 signaling pathway by transcriptome analysis. (A) Overlap of identified DEGs (deferentially expressed genes) in two independent biological replica. (B) KEGG pathway enrichment of DEGs is common in two biological replicates. *P* values were calculated using the following formula: 
$$P=1-\sum_{i=0}^{M-1}\frac{\left({}_{i}^{M}\right)\left({}_{n-i}^{N-M}\right)}{\left({}_{n}^{N}\right)}$$
where *N* is the number of all genes with that KEGG annotation, *n* is the number of DEGs in *N*, *M* is the number of all genes annotated to specific pathways, and *m* is the number of DEGs in *M*. The calculated *P* value goes through FDR correction, taking the corrected *P* value (the q-value) of ≤0.05 as a threshold. Download FIG S3, TIF file, 1.4 MB.Copyright © 2019 Wang et al.2019Wang et al.This content is distributed under the terms of the Creative Commons Attribution 4.0 International license.

10.1128/mSphere.00259-19.4 FIG S4 Tryptophan metabolism pathway (ko00380) enriched in DEGs in a comparison of the WT and *ssagc1*Δ mutant. Downregulated genes are shown in green boxes. This image is from the KEGG pathway database (http://www.kegg.jp/) developed by Kanehisa Laboratories, and reproduction is allowed for academic purposes. Download FIG S4, TIF file, 0.08 MB. Copyright © 2019 Wang et al. 2019 Wang et al. This content is distributed under the terms of the Creative Commons Attribution 4.0 International license.

10.1128/mSphere.00259-19.5 FIG S5 MAPK signaling pathway (ko04011) enriched in DEGs in a comparison of the WT and *ssagc1*Δ mutant. Downregulated genes are shown in green boxes. This image is from KEGG pathway database (http://www.kegg.jp/) developed by Kanehisa Laboratories, and reproduction is allowed for academic purposes. Download FIG S5, TIF file, 0.04 MB. Copyright © 2019 Wang et al. 2019 Wang et al. This content is distributed under the terms of the Creative Commons Attribution 4.0 International license.

10.1128/mSphere.00259-19.6 FIG S6 Schematic representation of the developmental and/or metabolism pathways containing the DEGs identified in transcriptional profiling. This scheme mainly contains the MAPK signaling pathway in response to fungal pheromone perception and leading to *a* and *b* mating locus induction via Prf1 transcription factor, as well as tryptophan metabolism through the Ehrlich pathway. Downregulated genes are shown in green boxes, while upregulated genes are shown in red boxes. The aromatic chemical compounds in red boxes were able to restore mating/filamentation in the *ssagc1*Δ mutant, while the ones in black boxes were not. Download FIG S6, TIF file, 1.7 MB. Copyright © 2019 Wang et al. 2019 Wang et al. This content is distributed under the terms of the Creative Commons Attribution 4.0 International license.

10.1128/mSphere.00259-19.10 DATA SET S1 List of DEGs (*P* ≤ 0.05) and the enriched KEGG terms in the *ssagc1*Δ mutant compared to the wild-type *MAT-1* strain. The file contains five worksheets or tabs: DEGs of Control versus T-1 (*P* ≤ 0.05), DEGs of Control versus T-2 (*P* ≤ 0.05), overlapped DEGs of two sets, query to map enrichment, and map to query enrichment. T represents ssagc1-deletion mutant, and -1 and -2 represent repeat 1 and repeat 2, respectively. Download Data Set S1, XLS file, 0.7 MB. Copyright © 2019 Wang et al. 2019 Wang et al. This content is distributed under the terms of the Creative Commons Attribution 4.0 International license.

### Tryptophan metabolites restored *ssagc1*Δ mating/filamentation.

**FIG 5:**
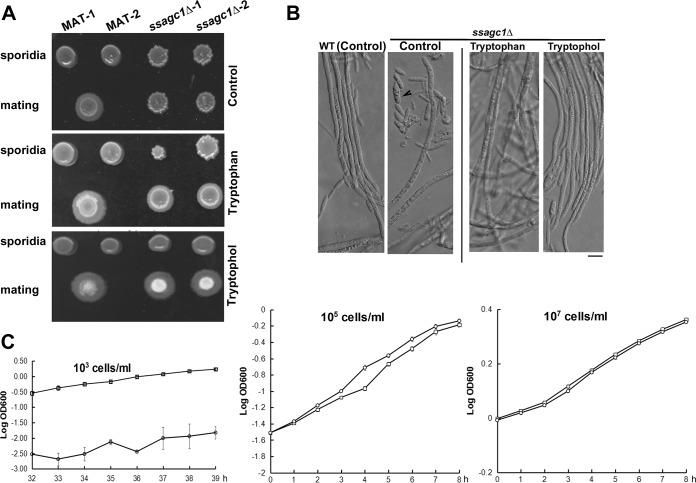
Tryptophan metabolites restored *ssagc1*Δ mating/filamentation. (A) Mating/filamentation of WT and *ssagc1*Δ mutant was assessed on PDA solid medium supplemented with tryptophan (3.3 μM) or tryptophol (25 μM). Photographs were taken 3 days postinoculation. The control was a untreated culture. The top rows of each of the three panels show sporidial colonies; the bottom rows show mating of the WT or mutant sporidia (in *MAT-1* background) with the WT *MAT-2* sporidia. (B) Microscopic imaging of hyphae formed after sexual mating, in the WT or *ssagc1*Δ mutant, supplemented with tryptophan (3.3 μM) or tryptophol (25 μM). For the control, no chemical supplement was added. Images were taken 48 h postinoculation. Bar = 1 mm. (C) Effect of tryptophol on *S. scitamineum* sporidial growth. A 24-h culture of *S. scitamineum* sporidia was diluted into YePS liquid medium at 10^3^, 10^5^, or 10^7^cells per ml in the presence (circles) or absence (squares) of 25 μM tryptophol. The number of cells at each specific time point was acquired by measuring the OD absorption at 600 nm. The growth curve for each culture was prepared by plotting the logarithmic values of OD600 versus incubation time.

Our transcriptional profiling results suggest that SsAgc1 plays a key role in regulation of tryptophan metabolism, which produces a known yeast quorum-sensing molecule tryptophol (13), and phytohormone indoleacetate (IAA). Thus, we tested the effects of tryptophan, tryptophol, and IAA on *S. scitamineum* mating/filamentation. Our results showed that tryptophan could partially restore *ssagc1*Δ filamentation only at a very low concentration (3.3 μM; Fig. 5A), and tryptophol (25 μM) could fully restore *ssagc1*Δ filamentation (Fig. 5A). Microscopic observation was performed to closely examine the hyphal morphology in the WT and *ssagc1*Δ mutant. We observed abundant nonmating sporidia in the *ssagc1*Δ mutant without any treatment (Fig. 5B, black arrowhead), and hyphae were formed occasionally. In contrast, long and smooth hyphae were formed in the WT cultured under the same conditions (Fig. 5B). Treatment with tryptophan or tryptophol promoted hyphal formation in the *ssagc1*Δ mutant, which were morphologically identical to WT hyphae (Fig. 5B). However, IAA could not restore *ssagc1*Δ mating/filamentation (Fig. S7A). In summary, we found that tryptophan and tryptophol could differentially promote *S. scitamineum* mating/filamentation at various concentrations and were able to at least partially restore the defects of *ssagc1*Δ in mating/filamentation.

10.1128/mSphere.00259-19.7 FIG S7 (A) Effect of IAA on *S. scitamineum* mating/filamentation. Mating/filamentation of the WT and *ssagc1*Δ mutant was assessed on PDA solid medium supplemented with various concentrations of IAA. Photographs were taken 3 days postinoculation. (B) Detection of tryptophol from the crude extract of sporidial culture by LC-MS in the positive ionization mode, SIM. The retention time (RT) for trypotophol is around 3.98 to 4.01 min. (C) Standard curve for trytophol (Sigma-Aldrich, V900672) concentration by plotting the peak area (relative abundance) to the diluted tryptophol of 10 to 100 nM. Download FIG S7, TIF file, 1.3 MB. Copyright © 2019 Wang et al. 2019 Wang et al. This content is distributed under the terms of the Creative Commons Attribution 4.0 International license.

### Tryptophol production during *S. scitamineum* sporidial budding growth.

As tryptophol could promote hyphal formation in the WT strain and fully restore the defects of *ssagc1*Δ in mating/filamentation, we suspected that it may also act as a quorum-sensing molecule in *S. scitamineum*. To prove that, we examined the relationship of tryptophol with *S. scitamineum* cell density. Interestingly, the addition of tryptophol (25 μM) displayed a growth-promoting activity on WT sporidia when the initial sporidial density was adjusted to 10^5^ cells/ml, but such growth-promoting property was not observed when the initial inoculation of WT sporidia was increased to 10^7^ cells/ml (Fig. 5C). However, when the initial sporidial density was as low as 10^3^ cells/ml, growth (budding) was suppressed by tryptophol (Fig. 5C).

**FIG 6:**
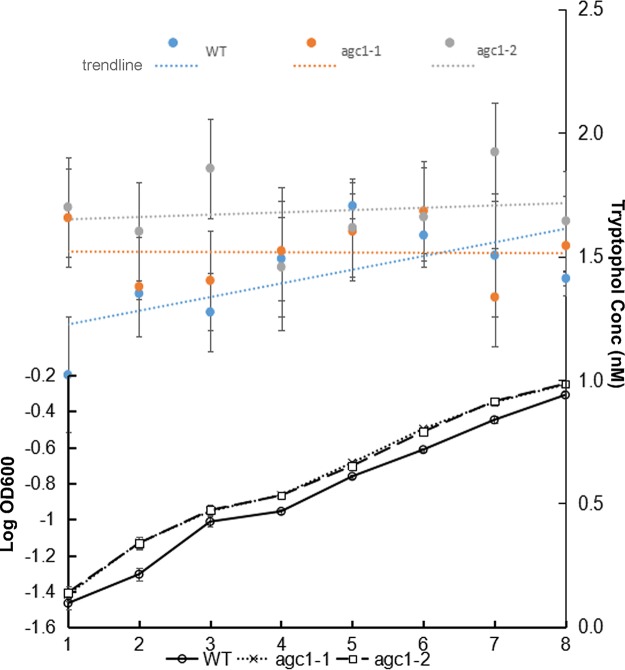
Detection of tryptophol in the WT and the *ssagc1*Δ mutant during sporidial growth. Sporidia from the wild-type strain or *ssagc1*Δ mutants (two independent strains) were cultured in the liquid YePS medium at 28°C, with shaking at 200 rpm, until an OD600 of 2.0 was reached. The sporidia were then diluted in 1,000 ml fresh YePS liquid medium, adjusting to a cell density of 10^5^ cells per ml, and cultured for another 8 h under the same conditions. The number of cells for each hour was acquired by measuring the OD absorption at 600 nm. The growth curve for each culture was prepared by plotting the logarithmic values of OD600 versus incubation time. For every hour, 10 ml of sporidial culture was extracted with 1.5× volume of ethyl acetate, and dried by rotary evaporation (EYELA, OSB-2100). The crude extracts were then dissolved in methanol and filtered with 0.22-μm PVDF membrane (Nylon6) for HR-ESI-MS analysis to determine the concentration of tryptophol. Means ± standard deviations (error bars) are shown for the tryptophol concentrations.

We further measured tryptophol production in *S. scitamineum* sporidial growth (budding) by high-resolution electrospray ionization mass spectrometry (HR-ESI-MS). Our results showed that in the first 8-h growth, WT sporidia increased steadily, with concurrent increase in tryptophol production (Fig. 6 and Fig. S7B and C), confirming that tryptophol production is dependent on cell density in *S. scitamineum*. Sporidial growth of two *ssagc1*Δ mutants was slightly faster than that of the WT. However, tryptophol production in these two mutants did not show significant differences compared to that in the WT sporidia (Fig. 6); therefore, loss of *SsAGC1* did not cause changes in tryptophol production.

## DISCUSSION

In multicellular eukaryotes, including fungi, AGC kinases have been reported to link various signaling events to regulate cell growth, development, and aging. The evolutionarily conserved TORC1-Sch9 in S. cerevisiae was shown to link nutrient sensing and aging (33), and it likely acts to provide functional compensation to cAMP/PKA-deficient yeast mutants (34). The cAMP/PKA pathway downstream kinase Rim15 cross-talks with the TORC1-Sch9 pathway in S. cerevisiae and Yarrowia lipolytica to regulate yeast dimorphic transition (33, 35). Homologs of the AGC kinase Sch9 have been identified in several pathogenic fungi, including Aspergillus nidulans (36), Aspergillus fumigatus (37), and F. graminearum (38), where it contributes to fungal virulence independently or via partial overlap with the cAMP/PKA pathway. In smut fungi, however, the AGC signaling pathway has not been identified or characterized.

Our present study identified an AGC kinase-encoding gene in *S. scitamineum*, named *SsAGC1*. The predicted polypeptide encoded by *SsAGC1* possesses the conserved catalytic domains while lacking an N-terminal lipid binding domain and a C-terminal regulatory domain containing a consensus hydrophobic sequence, as reported in ascomycetous AGC kinases. Instead, it contains a PAS domain in front of the catalytic domains, and a response regulatory (RR) domain of the CheY-like superfamily at the C terminus. Our genetic complementation was performed using a truncated polypeptide containing all the conserved domains (2082 to 4170 aa), which could fully restore the mating/filamentation defect in the *ssagc1*Δ mutant, indicating that this region is sufficient for SsAgc1 function in *S. scitamineum* mating/filamentation. The long N-terminal peptide (1 to 2081 aa) of SsAgc1 contains no identified domains, but the gene locus encoding this region was shown to be transcribed, in our previous high-throughput RNA sequencing (RNA-seq) data (27).

The presence of a PAS domain in the middle of SsAgc1 indicates that it may also be a PAS kinase, which is broadly evolutionarily conserved among eukaryotes, but not in C. elegans (32, 39). S. cerevisiae contains several PAS kinases, including Rim15, and they function in nutrient sensing, glucose homeostasis, and/or oxidative stress tolerance (31, 32, 40). However, the molecular mechanisms of PAS kinase regulation and function are largely unknown. There have not been reports on any function of PAS kinases in fungal filamentation/dimorphic switch. Our study showed that the filamentation defect of the *ssagc1*Δ mutant could be restored by culturing the fungus under nitrogen starvation conditions; therefore, it may represent the first PAS kinase essential for proper filamentation in the pathogenic fungus *S. scitamineum* in a nutrient-dependent manner.

The *ssagc1*Δ mutants could not mate/filament when mixed with the WT sporidia of the opposite mating type, in which a wild-type copy of *SsAGC1* gene was present. We inferred that SsAgc1 may predispose sporidia to mating, and the loss of SsAgc1 may make sporidia unable to respond to pheromone-induced sexual mating, even though the sporidia of the other mating type are functional.

Our results showed that deletion of *SsAGC1* led to upregulation of the *TYNA-1* gene and downregulation of the *amiE* gene. Production of the potential fungal quorum-sensing molecule tryptophol and phytohormone IAA both rely on the tryptophan-dependent biosynthesis pathway and share many intermediate metabolites (41–43). However, we noticed that tryptophol and IAA had different effects on *S. scitamineum* differentiation and morphogenesis. The addition of tryptophan or tryptophol could effectively promote *S. scitamineum* mating/filamentation and at least partially restore *ssagc1*Δ defects, while the addition of the phytohormone IAA could not. We also tested the effect of another aromatic alcohol, tyrosol, and it was not able to restore *ssagc1*Δ mating/filamentation (data not shown). Tryptophol might act as a potential quorum-sensing compound, as it shortened the lag phase of WT haploid sporidia grown in liquid culture, dependent on initial cell density. WT sporidia displayed a trend of cell density-dependent production of endogenous tryptophol. We speculate that *S. scitamineum* may manipulate IAA biosynthesis pathway to fungal QS molecule production as a part of its colonization strategy especially during its *in planta* growth stage. Detour of IAA biosynthesis or transport has recently been reported in S. reilianum, the closest smut relative of *S. scitamineum* (44, 45).

Overall, our study suggests a connection between AGC kinase and tryptophol production and/or signaling, but at present, we are not clear whether, or how, SsAgc1 regulates the responses to quorum sensing. There are also several interesting questions remaining unanswered in our work, such as whether SsAgc1 directly interacts with the signal molecule(s), given the presence of a PAS domain. The C-terminal RR domain implies nuclear translocation and direct transcriptional regulation of downstream gene expression by SsAgc1, pending further verification. Overall, we believe our work represents the first report on the AGC signaling pathway in *S. scitamineum* growth and differentiation. Furthermore, SsAgc1 signaling may integrate cell density, nutrient availability, and fungal mating/filamentation.

## MATERIALS AND METHODS

### Growth conditions and fungal strains used in this study.

Teliospores of sugarcane smut collected from the fields in Guangdong province of China (21°12′ 36′′ N; 101°10′ 12′′ E) by Yan et al. (1) were maintained in Lian-Hui Zhang’s lab, and the *MAT-1* or *MAT-2* haploid sporidia isolated from these teliospores were used in this study. The culture medium used in this study include YePSA medium (1% yeast extract, 2% peptone, 2% sugar, 1.5% agar), YePS liquid medium (1% yeast extract, 2% peptone, 2% sugar [pH 7.0]), YePS soft medium (1% yeast extract, 2% peptone, 2% sugar, 0.65% agar), YePSS medium (1% yeast extract, 2% peptone, 2% sugar, 18.17% d-sorbitol, 2% agar), PDA **(**HB0233-12; Beijing Dingguo) medium, and MM-N medium (1% glucose, 0.15% KH_2_PO_4_, 0.05% MgSO_4_, 0.05% KCl, 1,000× dilution of TES [2.2% ZnSO_4_, 1.1% H_3_BO_3_, 0.5% MnCl·4H_2_O, 0.5% FeSO4·7H_2_O , 0.17% CoCl_2_·6H_2_O, 0.16% CuSO_4_·5H_2_O, 0.13% NaMoO_4_·2H_2_O, 5% NaEDTA·2H_2_O] pH 6.5). For the mating/filamentation assay, equal volumes of haploid sporidia of *MAT-1* or *MAT-2* were mixed, plated on the solid medium, and kept in dark at 28°C incubator for 2 to 3 days before photographing. For the growth assay, wild-type (*MAT-1*) and *ssagc1*Δ mutant *S. scitamineum* sporidia were first cultured in 5 ml of YePS liquid medium at 28°C with shaking at 200 rpm for 24 h. Such cultured sporidia were then diluted to 200 ml fresh YePS liquid medium, adjusting to a cell density of 10^5^ cells per ml, and cultured for another 8 h under the same conditions. Measurement of the optical density at 600 nm (OD600) with a spectrophotometer (NanoDrop 2000C; Thermo Fisher Scientific) was performed hourly to monitor the yeast-like (budding) growth of wild-type or mutant strains, with or without the addition of chemical reagents as described above in Results.

### Characterization of the SsAgc1 sequence.

The deduced protein sequence of SsAgc1 was analyzed using the Compute pI/*M*_w_ tool (http://web.expasy.org/compute_pi/) to determine the theoretical isoelectric point (pI) and molecular weight (MW), and interPro-Scan (http://www.ebi.ac.uk/interpro/) to identify domains. A phylogenetic tree was constructed using a neighbor-joining method following alignment with other protein kinases, using COBALT (constraint-based multiple alignment tool; https://www.ncbi.nlm.nih.gov/tools/cobalt/cobalt.cgi?CMD=Web).

### Chemical compounds.

Tryptophan (catalog no. DH357-2; Beijing Dingguo), tryptophol (catalog no. V900672; Sigma-Aldrich), and IAA (catalog no. DH171-5; Beijing Dingguo) were used in this study.

### Nucleic acid-related manipulation.

Fungal genomic DNA was extracted using a HP Fungal DNA kit (catalog no. D3195-01; Omega). PCR amplification was performed using Phusion high-fidelity DNA polymerase (lot 00528748; Thermo Fisher Scientific). DNA fragment elution was performed using Gel Extraction kit (catalog no. D2500-02; Omega) and/or Cycle Pure kit (catalog no. D6492-02; Omega). In the Southern blot assay, NEB restriction enzyme ClaI (R0197V) was used for digestion of genomic DNA. DIG-High Prime DNA Labeling and Detection Starter kit I (catalog no. 11745832910; Roche) was used for labeling of PCR-amplified fragments as probes. Amersham Hybond TM-N+ (catalog no. RFN303B; GE Healthcare) membrane was used for blotting. DIG-High Prime DNA Labeling and Detection Starter kit I (catalog no. 11745832910; Roche) was used for labeling of PCR-amplified fragments as probes. NBT/BCIP stock solution (catalog no. 11681451001; Roche) was used for probed band detection. For total RNA extraction, Qiagen RNeasy plant minikit (catalog no. 74104) was used. For removing contaminating DNA from RNA preparations, Ambion TURBO DNA-free kit (catalog no. AM1907; Invitrogen) was used. TransScript First-Strand cDNA Synthesis Super Mix (catalog no. AT301-02; Transgen) was used for cDNA synthesis. For quantitative real-time PCR (qRT-PCR), we used PowerUp SYBR green master mix (catalog no. A25742: Applied Biosystems) with the primers as listed in Table S1 in the supplemental material, and the reaction was run on QuantStudio 6 Flex Real-Time PCR system (Thermo Fisher Scientific).

10.1128/mSphere.00259-19.8 TABLE S1 Selected genes for transcriptional profiling for the WT and *ssagc1*Δ mutant. Download Table S1, DOCX file, 0.01 MB. Copyright © 2019 Wang et al. 2019 Wang et al. This content is distributed under the terms of the Creative Commons Attribution 4.0 International license.

### Generation of deletion or complementation constructs.

Construction of two fragments for the replacement of *SsAGC1* gene by the *HPT* (Hyg^r^) gene follows the strategy described previously (46, 47). The flanking DNA (1 kb 5′ and 3′) of the *SSAGC1* gene was PCR amplified using WT *S. scitamineum* genomic DNA as the template, and the *HPT* gene with plasmid pEX2 (1) as the template. The 5′- and 3′-flanking sequences, fused with two half partially overlapping *HPT* sequences, were generated by fusion PCR and verified by sequencing. For constructing genetic complementation of the *SsAGC1* gene, the *SsAGC1* open reading frame (ORF) sequence from +7860 to +14163 (stop codon) was PCR amplified with the forward primer containing a start codon ATG (Table S2) and inserted into our complementation plasmid pEX2-Zeo-dsRed. The resultant plasmid was named EX2-Zeo-AGC1. A constitutive promoter *G3PD Prom* fragment was PCR amplified and inserted in the pEX2-Zeo-AGC1 plasmid in front of the *SsAGC1* ORF to form plasmid pEX2-Zeo-G3PD Prom-AGC1. This complementation plasmid was transformed into the *ssagc1*Δ-1 mutant, and the complemented strains were first screened by resistance to 100 μg/ml zeocin (catalog no. R25001; Invitrogen) and then verified by PCR amplification of zeocin fragment and qRT-PCR analysis of *SsAGC1* gene expression. All the primers used for construction or verification of deletion or complementation strains are listed in Table S2.

10.1128/mSphere.00259-19.9 TABLE S2 List of primers used for gene deletion/complementation. Download Table S2, DOCX file, 0.01 MB. Copyright © 2019 Wang et al. 2019 Wang et al. This content is distributed under the terms of the Creative Commons Attribution 4.0 International license.

### PEG-mediated protoplast transformation.

Polyethylene glycol (PEG)-mediated protoplast transformation follows the established protocol (47) modified as follows. Enzyme digestion of the wild-type *MAT-1* sporidia was performed using the lysing enzyme (catalog no. L1412; Sigma) dissolved in SCS solution (20 mM trisodium citrate and 1 M d-sorbitol [pH 5.8]) to reach the final concentration of 0.02 g/ml, incubated at 28°C for about 30 min. A 40% PEG solution was prepared by dissolving 4 g PEG 3350 (catalog no. 202444; Sigma-Aldrich) in 10 ml STC solution (10 mM Tris-HCl, 1 M d-sorbitol, and 100 mM CaCl_2_ [pH 7.5]). The PCR-amplified fragments of 1 to 5 μg, 1 μl heparin solution (15 mg/ml) (Beijing Dingguo, DH157) and the protoplasts were mixed and incubated with 40% PEG solution on ice for 10 min. The protoplasts were regenerated on the three-layer regeneration medium: one layer of YePS soft medium on top of two lays of YePSS medium, with only the bottom YePSS layer containing 400 μg/ml hygromycin B (Calbiochem, CAS: 53-84-9).

### RNA-seq and transcriptome analysis.

High-throughput RNA sequencing (RNA-seq) and transcriptome analysis were performed by Gene Denovo company (Guangzhou, China) using the reported protocols (27) with the following minor modification. Short reads were mapped to the complete genome of *S. scitamineum* (ftp://ftp.ensemblgenomes.org/pub/fungi/release-38/fasta/fungi_basidiomycota1_collection/sporisorium_scitamineum/dna/) using Tophat (48, 49).

### Staining protocols and microscopy.

Calcofluor white (catalog no. 18909; Sigma-Aldrich) was used at 3 μg/ml (in 100 mM Tris-HCl buffer [pH 9.0] containing Triton X-100 at 1:1,000) to visualize cell wall and septa of the sporidia of the respective strains. To stain nuclei, *S. scitamineum* sporidia were incubated in 1 μg/ml Hoechst (catalog no. 33258; Invitrogen-Molecular Probes) solution for 15 min at room temperature, followed by a thorough wash with sterile water. Epifluorescence microscopy and imaging were performed using an Axio Observer Z1 microscope (Zeiss, Jena, Germany) equipped with an sCMOS camera (PCO Edge, Kelheim, Germany).

### Determination of intracellular cAMP concentration.

Sporidia from wild-type *ssagc1*Δ, and *ssuac1*Δ strains were cultured in the liquid YePS medium at 28°C, with shaking at 200 rpm, until an optical density at 600 nm of 2.0 was reached. A total of 10^7^ sporidia of each strain were aliquoted and streaked on YePSA medium for a further 24-h culture in the dark at 28°C, before they were harvested and ground with liquid nitrogen. The ground samples were subject to quantitative determination of cyclic AMP using a cAMP Enzyme Immunoassay kit (catalog no. CA201; Sigma-Aldrich) following the manufacturer’s instructions.

### Determination of tryptophol concentration in sporidial culture.

For high-resolution electrospray ionization mass spectrometry (HR-ESI-MS) determination of tryptophol concentration, 10-μl portions of the prepared samples were analyzed with HR-ESI-MS (Q-Exactive Focus; Thermo Fisher Scientific), used by acetonitrile (ACN)-H_2_O (5:95 to 95:5, 0 to 4 min: 5:95, 4 to 5 min). The HR-ESI-MS showed a quasi-molecular ion peak at *m/z* 162.09134 (tryptophol+H^+^) [M + H]^+^. To make a standard curve, tryptophol (catalog no. V900672; Sigma-Aldrich) stock of 100 mM was diluted to 20 nM, 40 nM, 60 nM, 80 nM, and 100 nM, and analyzed by HR-ESI-MS with the same settings as mentioned above.

### Pathogenicity assay.

The susceptible sugarcane variety ROC22 was inoculated with mixed fungal sporidia from WT or mutant combination (of opposite mating type) by injecting seedlings with four to five leaves. The disease symptoms were photographed at 90 to 180 days postinoculation (dpi).

### Statistical analysis.

Statistical significance of the expression data was determined at *P* < 0.01 (**) and *P* < 0.05 (*) using Student’s *t* test.

### Data availability.

All the data present in this article are available in the main body or supplemental material.
