# Supplementary figures and images for: The AGC Kinase SsAgc1 Regulates Sporisorium scitamineum Mating/Filamentation and Pathogenicity
Source: mSphere. 2019 May 29;4(3):e00259-19. doi: 10.1128/mSphere.00259-19 (PMC6541736; doi:10.1128/mSphere.00259-19)

Figure S1

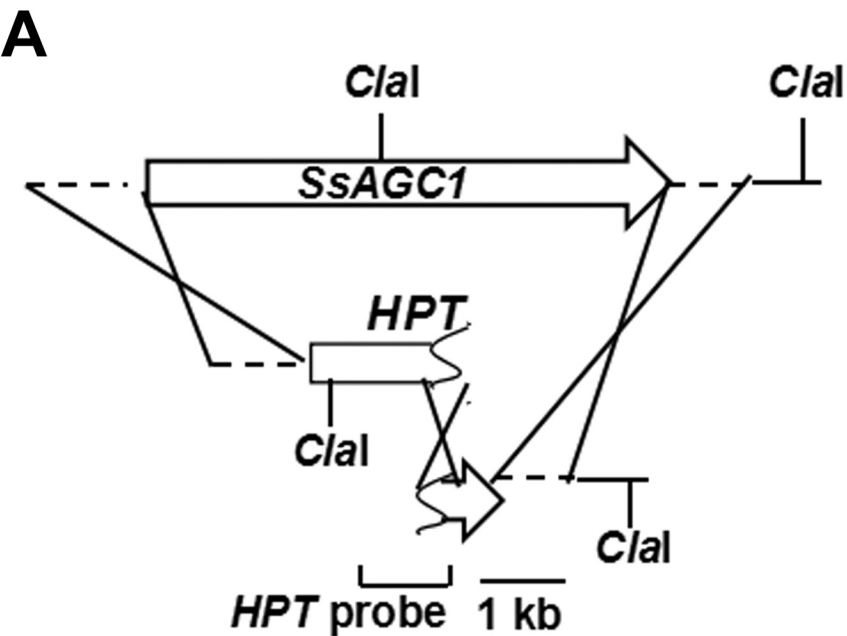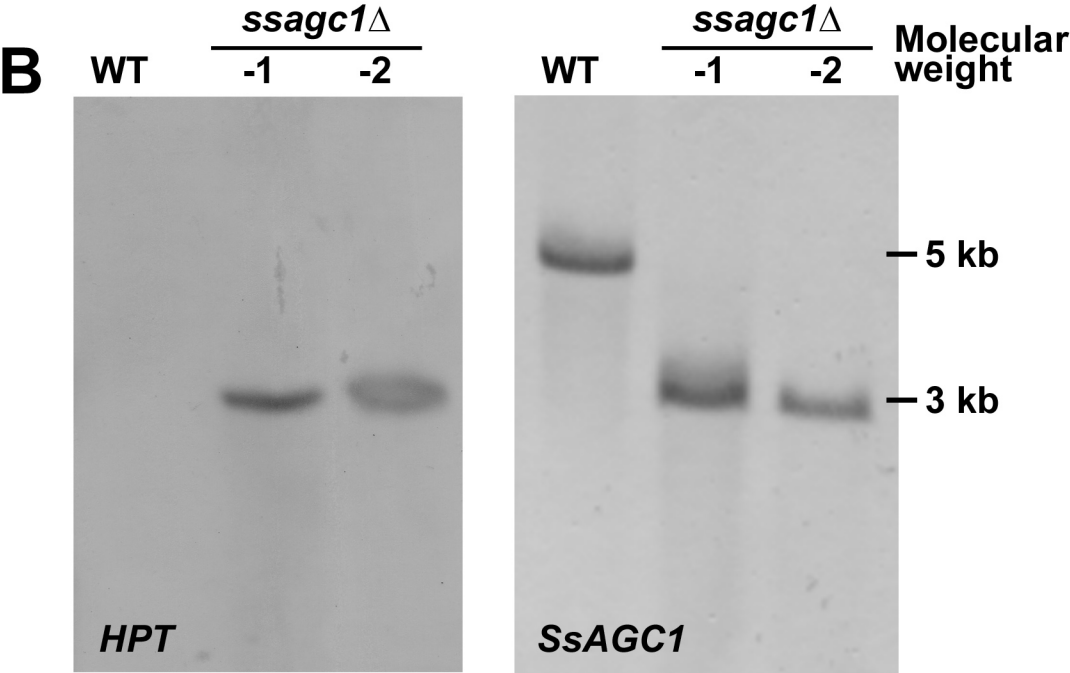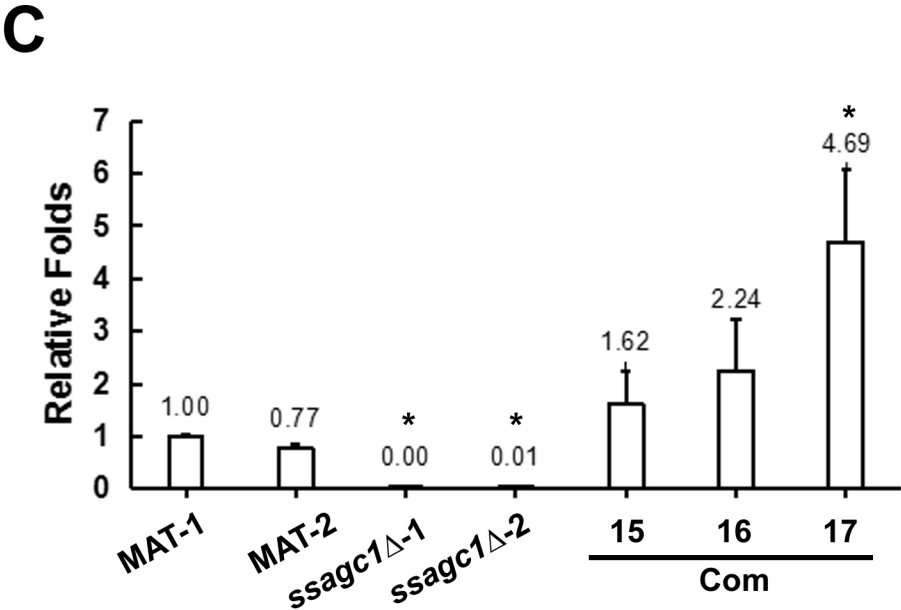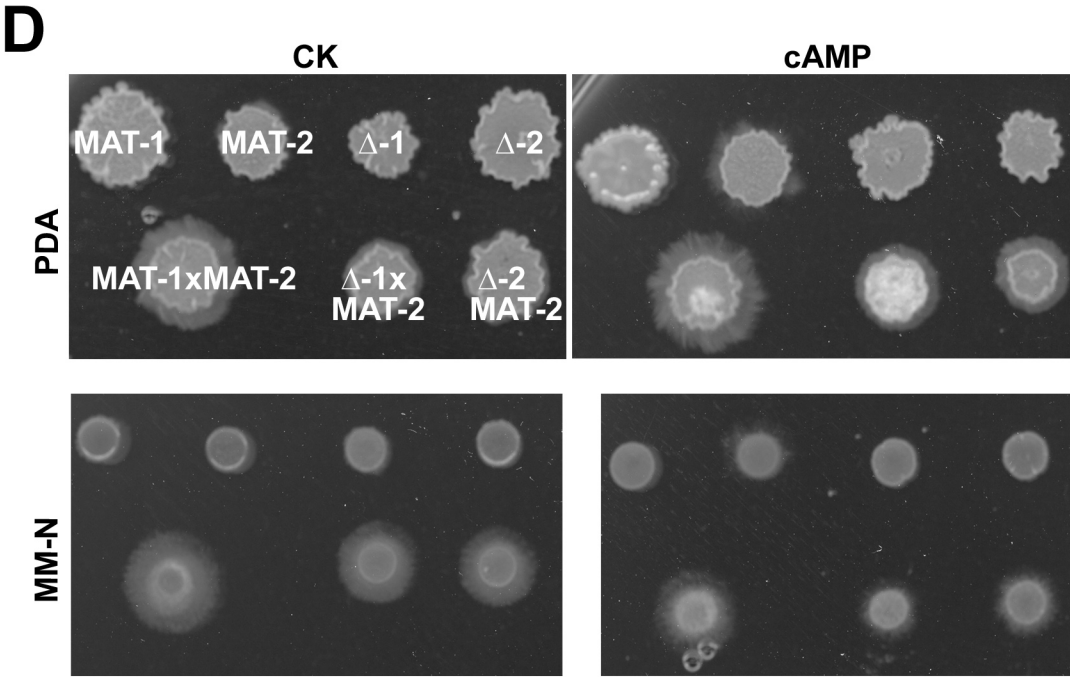

Supplement: FIG S1 [file mSphere.00259-19-sf001.pdf]

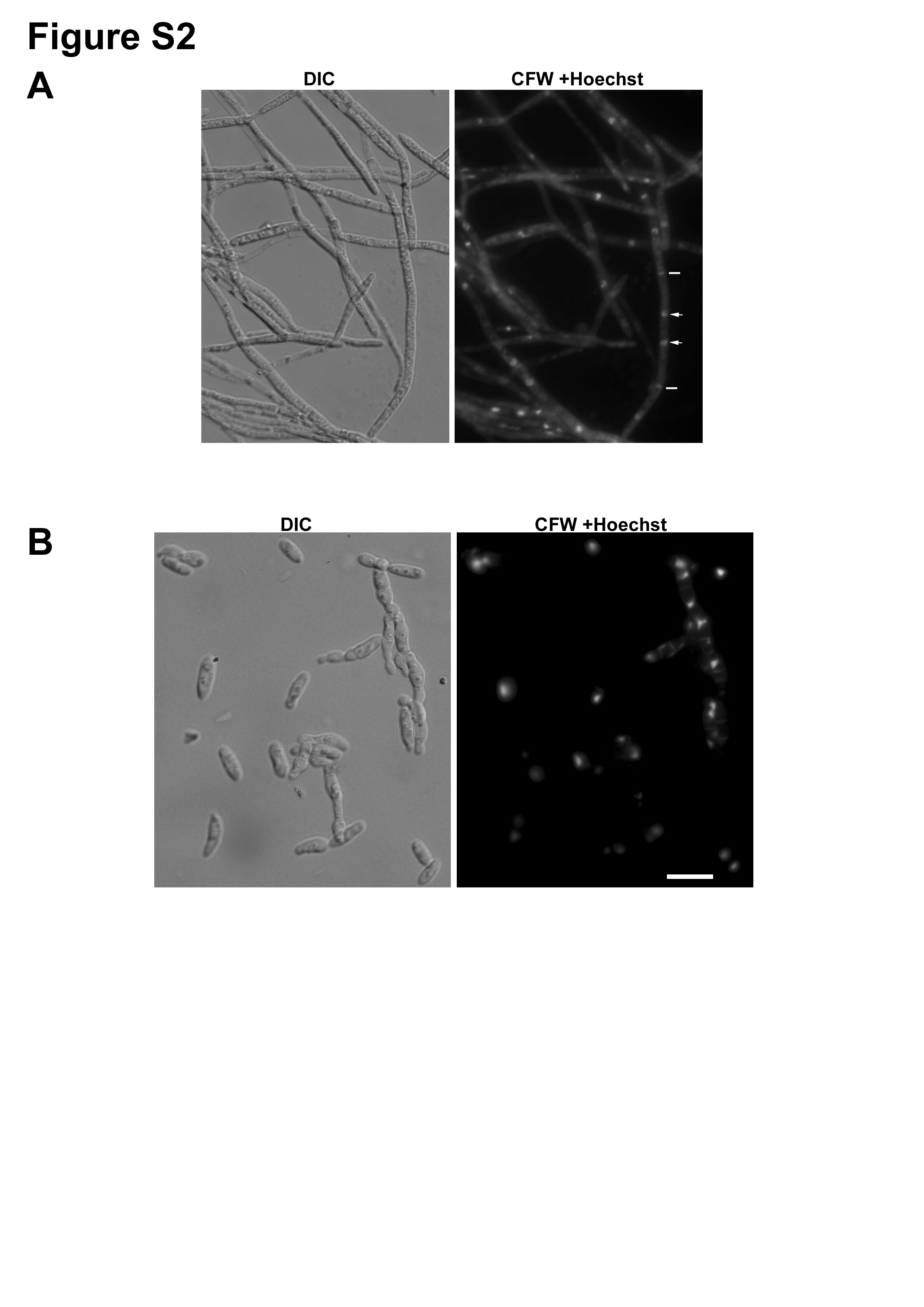

Supplement: FIG S2 [file mSphere.00259-19-sf002.tif]

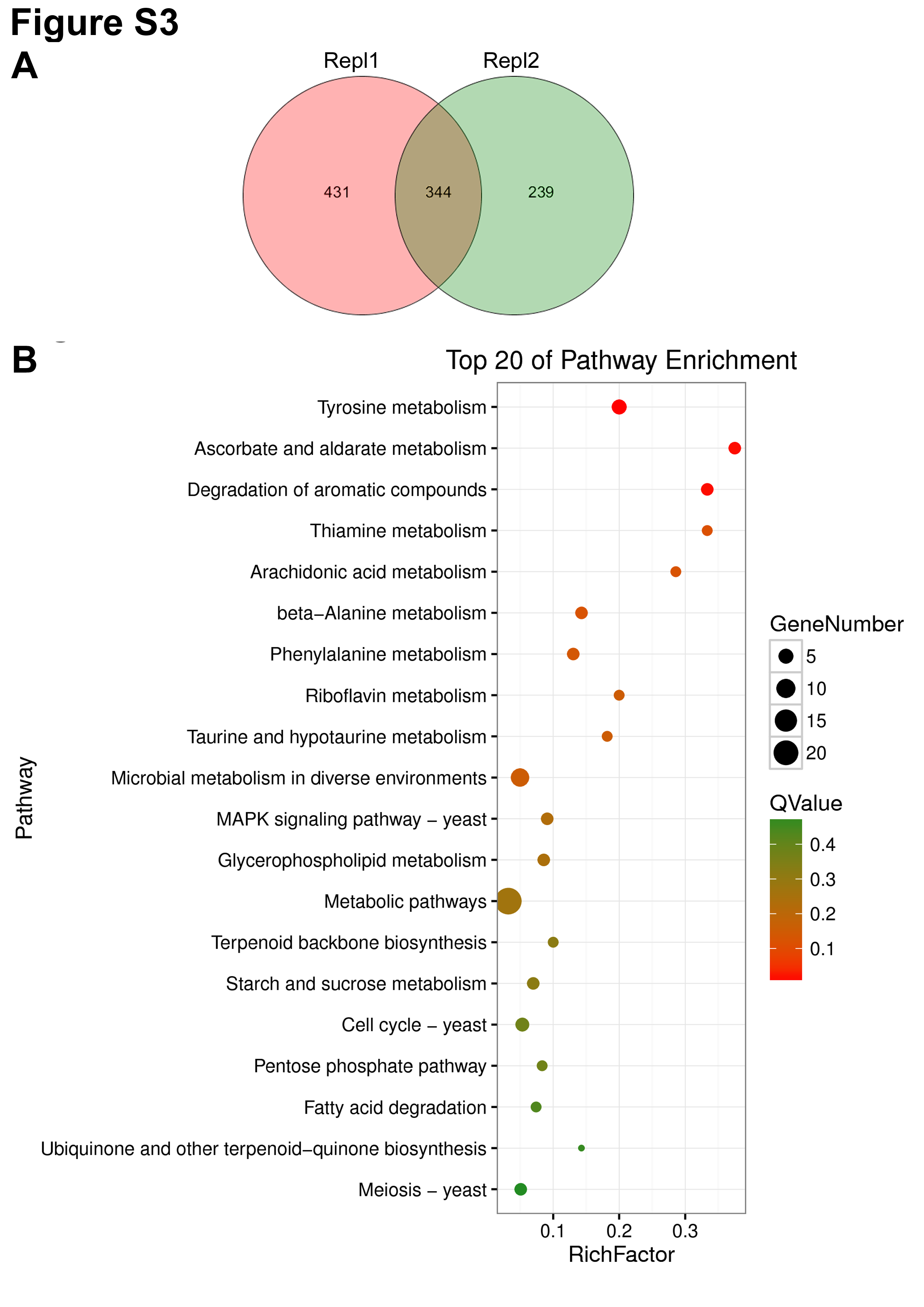

Supplement: FIG S3 [file mSphere.00259-19-sf003.tif]

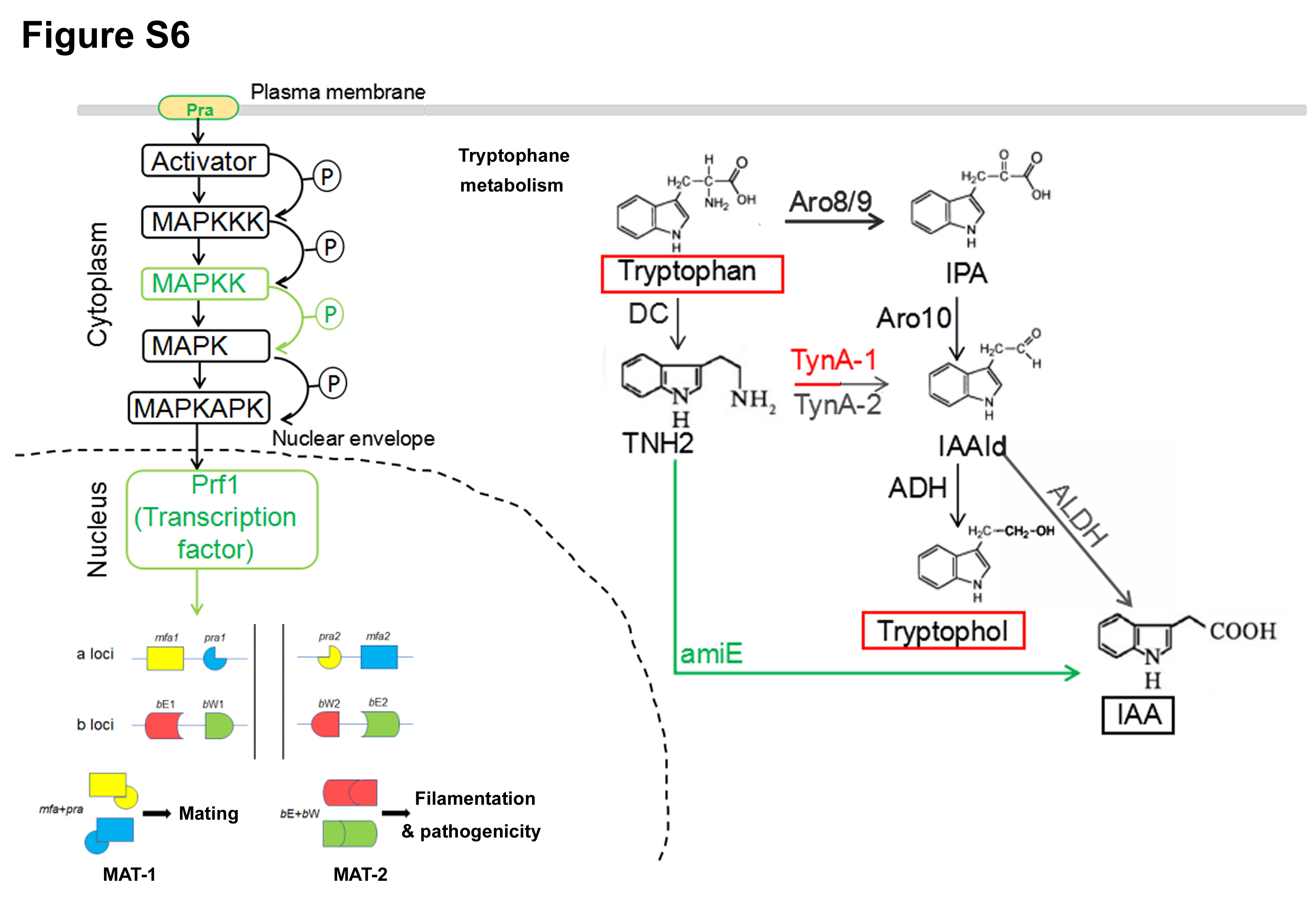

Supplement: FIG S6 [file mSphere.00259-19-sf006.tif]

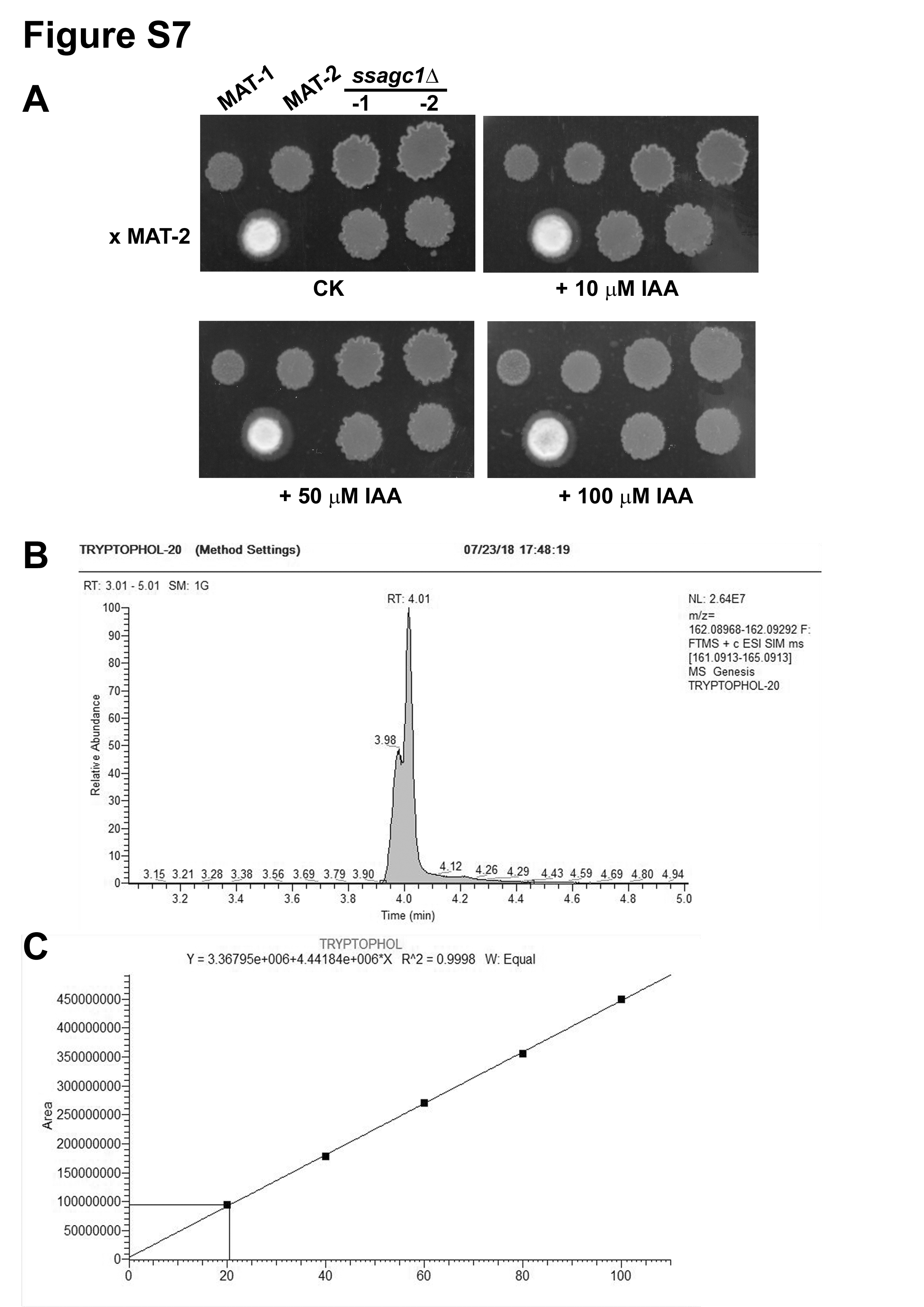

Supplement: FIG S7 [file mSphere.00259-19-sf007.tif]
