# Supplementary material for: The AGC Kinase SsAgc1 Regulates Sporisorium scitamineum Mating/Filamentation and Pathogenicity
Source: mSphere. 2019 May 29;4(3):e00259-19. doi: 10.1128/mSphere.00259-19 (PMC6541736; doi:10.1128/mSphere.00259-19)
Supplement: TABLE S1 [file mSphere.00259-19-st001.docx]

**Table S1.** Selected genes for transcriptional profiling between WT and *ssagc1*Δ.

| Genbank | Gene name | Sequences of qRT-PCR primers |
| --- | --- | --- |
| GenBank:  CP010914.1  857085-857204  (CDW95248.1) | *MFA1* | MFA1-For: 5’-ATGCTTTCCATCTTTACCCAGA-3’ |
|  |  | MFA1-Rev: 5’-GTGCAGCTAGAGTAGCCAAG-3’ |
| GenBank: LK056662.1  794925-795044 | *MFA2* | MFA2-For: 5’-CGTCCAGGCCATTGTTTCT-3’ |
|  |  | MFA2-Rev: 5’-TAGGCCACGGTGCAGTA-3’ |
| GenBank:  CP010914.1  859544-860833 | *PRA1* | PRA1-For: 5’-GGACGCTATCACCCAATCTTAC-3’ |
|  |  | PRA1-Rev: 5’-TCTCCAACATGGCAACACTC-3’ |
| CDU23176.1 | *PRA2* | PRA2-For: 5’-GAAGAGCCTCAGCCGTTATAC-3’ |
|  |  | PRA2-Rev: 5’-GGGTTCCCTTACTGAACCTTAG-3’ |
| CDW97668.1 | *bE* | bE-For: 5’-CCAACGACGAAAGCGCGACG -3’ |
|  |  | bE-Rev: 5’-GACTCTCTGCGAGCGGGCAT-3’ |
| CDS00151.1 | *bW* | bW-For: 5’-CGAGAAAGGCACACAACGTC-3’ |
|  |  | bW-Rev: 5’-CACCTTTTGGGGAGTTCCGA-3’ |
| CDW96669.1 | *PRF1* | PRF1-For: 5’-GTCGACCTCTTTCACGGATG-3’ |
|  |  | PRF1-Rev: 5’-CTCGCTTGGGAAAGGAGATG-3’ |
| CDS01951.1 | *ARO8* | ARO8-For: 5’-CCTGGTGTTGCGTTCATTCC-3’ |
|  |  | ARO8-Rev: 5’-CAAGCTCGGGCATCGTCTTA-3’ |
| CDW94109.1  CDR98564.1 | *ARO9* | ARO9-For: 5’-TCCGCACGAACCATCCTAAC-3’ |
|  |  | ARO9-Rev: 5’-AGGTCGAATGAGTCGCCTTG-3’ |
| CDW98745.1 | *TYNA-1* | TYNA-1 For: 5’-ACCAGTCCAACGGTAAGCAG-3’ |
|  |  | TYNA-1 Rev: 5’-TCTCCCAGAAATCGTTGGGC-3’ |
| CDW94194.1 | *TYNA-2* | TYNA-2 For: 5’-AAGGTTAACTCTGCCGCTCC-3’ |
|  |  | TYNA-2 Rev: 5’-GCTAATACCATGCCTCGGCT-3’ |
| CDS00690.1 | *amiE* | amiE-For: 5’-CAGCTGGAGTTGAGCGATGA-3’ |
|  |  | amiE-Rev: 5’-CCCACACAAGCTGCAAACTC-3’ |
| CDW96656.1 | *ACTIN* | Actin-For: 5’-CAGCTCGATGAAGGTCAAGAT-3’ |
|  |  | Actin-Rev: 5’-CACATCTGCTGGAAGGTAGAG-3’ |
| CDR87150.1 | *AGC1* | AGC1-For: 5’-CATCAATTCGCCTGCTTCCG-3’ |
|  |  | AGC1-Rev: 5’-CCTGGCGTTGCGGGATCTGA-3’ |
