# Supplementary material for: The AGC Kinase SsAgc1 Regulates Sporisorium scitamineum Mating/Filamentation and Pathogenicity
Source: mSphere. 2019 May 29;4(3):e00259-19. doi: 10.1128/mSphere.00259-19 (PMC6541736; doi:10.1128/mSphere.00259-19)
Supplement: TABLE S2 [file mSphere.00259-19-st002.docx]

**Table S2.** List of primers used for gene deletion/complementation.

| Name | Description | Primer sequences |
| --- | --- | --- |
| *Agc1-1For* | Deletion construction | 5’-AGAGACGCATCTGACGGTGAGCAGCAGC-3’ |
| *Agc1-1Rev* |  | 5’-TCAGCAAGATCTGGGCGAGTGCGAGCGCGAAA-3’ |
| *Agc1-2For* |  | 5’-TCGCACTCGCCCAGATCTTGCTGATAGGCAGG-3’ |
| pDNA*-Hpt-Rev* |  | 5’-GGTCAAGACCAATGCGGAGC-3’ |
| pDNA*-Hpt-For* |  | 5’-GCAAGACCTGCCTGAAACCG-3’ |
| *Agc1-2Rev* |  | 5’-TGTGACAGTCAGTAATTCGGGGGATCTGGAT-3’ |
| *Agc1-3For* |  | 5’-TCCCCCGAATTACTGACTGTCACACATAACCAAC-3’ |
| *Agc1-3Rev* |  | 5’-CCAAGGTTCTCCCGCATCTTCAGCGTTA-3’ |
| *Mfa1-For* | Verification | 5’-CGTCAGGGTCTTGACCACCTAGACAAG-3’ |
| *Mfa1-Rev* |  | 5’-GCGAAGGGCGCAGTTTTCAGTGGGCGT-3’ |
| *Agc1For* |  | 5’-GGCTGCTATGCCTATTGGTATTGT-3’ |
| *Agc1Rev* |  | 5’-TTGTACAATGTCTTGAGCTGGCTTCAGGTCTCG-3’ |
| *Hpt-For* |  | 5’-GCTGCGGCCGATCTTAGCCA-3’ |
| *Hpt-probe For* | Southern blot probe | 5’-ATGAAAAAGCCTGAACTCACCGC-3’ |
| *Hpt-probe Rer* |  | 5’-CTATTTCTTTGCCCTCGGACGAG-3’ |
| *Agc1-probe For* |  | 5’-CTGACTGTCACACATAACCAACGA-3’ |
| *Agc1-probe Rev* |  | 5’-CCAAGGTTCTCCCGCATCTTCAG-3’ |
| *AGC1ORF-F* | Complementation construction | 5’-cactcttccaccgatccATGTCGCTCTTTACTTCGCCGACCC-3’ |
| *AGC1ORF-R* |  | 5’-gtcCATgagctcggtacccggCGACAGCGATAACCTCTCAGCCAC-3’ |
| *G3PDp-F* |  | 5’-aggactgaactagtcgattgagatcttgctgataggcaggtttgct-3’ |
| *G3PDp-R* |  | 5’-gagctcggtacccggggatcggtggaagagtgttttggtttcgaaa-3’ |
